# Two-Dimensional “Nanotanks” Release “Gas Bombs” through Photodynamic Cascades to Promote Diabetic Wound Healing

**DOI:** 10.34133/bmr.0100

**Published:** 2024-10-29

**Authors:** Jiyuan Zou, Zhikang Su, Wen Ren, Yunxin Ye, Xuechao Yang, Tao Luo, Li Yang, Lvhua Guo

**Affiliations:** ^1^ Department of Endodontics, Affiliated Stomatology Hospital of Guangzhou Medical University, Guangdong Engineering Research Center of Oral Restoration and Reconstruction, Guangzhou Key Laboratory of Basic and Applied Research of Oral Regenerative Medicine, Guangzhou, Guangdong, People’s Republic of China.; ^2^ Department of Prosthodontics, School and Hospital of Stomatology, Guangdong Engineering Research Center of Oral Restoration and Reconstruction and Guangzhou Key Laboratory of Basic and Applied Research of Oral Regenerative Medicine, Guangzhou Medical University, Guangzhou, Guangdong, People’s Republic of China.

## Abstract

The emergence of multidrug-resistant (MDR) bacterial infections, particularly in diabetic wounds, represents a major challenge in clinical care due to their high mortality rate. Despite the continued use of antibiotics as the primary clinical treatment for diabetic wounds, there is an urgent need to develop antibiotic-free therapeutic strategies to combat MDR bacteria, given the limitations and resistance of antibiotics. In this study, a “nanotank”, MXene@MOF@CORM-401 (MMC), was designed to target bacteria. The basis of this approach is the combination of 2-dimensional transition metal carbides/carbon nitrides (MXene), metal–organic frameworks (MOFs), and carbon monoxide-releasing molecules (CORMs). MMCs exhibit photothermal and photodynamic properties upon irradiation with near-infrared laser. The photodynamic effect generates a substantial quantity of reactive oxygen species, which subsequently triggers the release of carbon monoxide in a “gas bombs”-like manner. In vitro and in vivo experiments have demonstrated that MMC is not only biocompatible but also exhibits robust antimicrobial properties and accelerates diabetic wound healing. Consequently, this innovative 2-dimensional “nanotank” represents a promising alternative to conventional antibiotic therapies for the treatment of MDR bacterial infections in the future.

## Introduction

According to the International Diabetes Federation, diabetes is a prevalent metabolic disease that is expected to affect approximately 537 million adults worldwide by 2021 [1]. The elevated glucose levels in diabetic patients create a favorable environment for bacterial proliferation and weaken the body’s natural defenses against infection [2]. This combination hinders effective bacterial clearance and prolongs wound healing in diabetic patients [3,4]. Currently, surgical debridement and antibiotic therapy are the primary approaches to diabetic wound care [5]. However, the misuse of antibiotics has led to a gradual increase in bacterial resistance, evolving into multidrug-resistant bacteria. Consequently, treatment has become more challenging, often necessitating the use of nonantibiotic strategies at higher concentrations or in combination therapy.

Gas therapy, a new type of treatment mediated by gas molecules, has attracted much attention from the medical community in recent years [6,7]. Unlike traditional antibiotic therapies, gas molecules do not induce bacterial resistance and therefore show unique advantages in combating drug-resistant bacteria [8]. In gas therapy, carbon monoxide (CO) plays a key role. CO inhibits bacterial respiration and energy supply by blocking electron transfer in the respiratory chain and the oxidative phosphorylation process in the bacterial cells, resulting in efficient bacterial killing [9]. Additionally, studies have demonstrated that small gas molecules can freely diffuse into bacterial biofilms and exert direct antibacterial effects [10,11]. Currently, carbon monoxide-releasing molecules (CORMs) have been considered as an antimicrobial agent [12–14]. Among them, CORM-401 is a CO-releasing molecule that is sensitive to oxidants. CORM-401 is capable of rapidly releasing CO in oxidative stress environments while remaining stable under normal physiological conditions [15]. However, high concentrations of carbon monoxide can cause damage to normal tissues if not properly managed [16]. Therefore, it is critical to develop a versatile delivery system that can precisely deliver and control the release of carbon monoxide. In addition, combining the controlled release of carbon monoxide with other therapeutic treatments to create a synergistic inhibitory effect will reinvigorate the development of gas therapy.

Compared to conventional drug administration, drug delivery systems can enhance the bioavailability of drugs [17–19]. For instance, scaffolds and hydrogels can increase the local drug concentration and achieve sustained release of the medication. Metal–organic frameworks (MOFs) are a class of materials formed by coordination bonds between metal ions or clusters and organic ligands, creating a network structure. They are favored for drug loading and release due to their high porosity and abundant active sites. In particular, zeolitic imidazolium framework 8 (ZIF-8), as a subclass of MOFs, is conducive to drug loading and release due to its high porosity and abundant active sites. It is worth mentioning that ZIF-8, as a new photocatalytic material, has shown remarkable effects in photodynamic therapy (PDT) [20–22]. The active site region on its surface can capture O_2_ and H_2_O molecules. After absorbing light energy, it interacts with oxygen-containing molecules and generates a large amount of reactive oxygen species (ROS) through catalytic oxidation reaction. In contrast, MXene, as a 2-dimensional nanomaterial, is composed of transition metal carbides or carbonitrides [23,24]. This material has an ultrathin structure, abundant active sites, and biodegradability [25]. Since MXene has local surface plasmon resonance effect, it shows strong absorption in the near-infrared (NIR) region and photothermal conversion efficiency. Therefore, MXene can exert the effect of photothermal therapy (PTT) and effectively kill bacteria through photothermal transformation [26]. Importantly, both PTT and PDT are independent of antibiotics, which greatly reduces the risk of causing bacterial resistance [27].

Based on these characteristics we have devised a type of “nanotank” (MXene@MOF@CORM-401 [MMC]) with the specific objective of targeting bacteria. Upon irradiation, the MMC exhibits photothermal and photodynamic properties, generating heat and ROS. The rapid generation of ROS initiates the release of a “gas bombs” (CO) from CORM-401, thereby establishing a synergistic trimodal antimicrobial system. The incorporation of MXene into the system results in a notable enhancement of the photothermal conversion efficiency, reaching 34.6%. Secondly, the negative group on MXene enhances the dispersion and stability of MOF. The ultrasmall particle size of MOF exhibits a very high specific surface area, a property that not only improves the stability and bioavailability of CORM-401 but also provides more active sites for the photocatalytic reaction, thus further enhancing the photocatalytic efficiency of the system [28]. It is noteworthy that the positive charge carried by the MOF endowed the system with the ability to target bacteria. The surface membrane structure of bacteria is rich in negatively charged phospholipids, which enables the MOF-modified nanosheets to be more easily captured by the bacteria. This provides the antimicrobial system with the ability to target bacteria [29,30]. This “nanotank” achieves efficient biofilm disruption and antimicrobial properties by forming ultrasmall-sized MOF (ZIF-8) containing CORM-401 on the MXene. CO gas molecules can penetrate inside the biofilm and directly contact the bacteria, whereas photothermal and photodynamic effects are effective in disrupting the biofilm structure, causing it to lose its integrity and functionality. This approach offers a promising alternative to antibiotic therapy for the treatment of diabetic wounds infected by drug-resistant bacteria.

## Materials and Methods

### Materials and cells

Ti_3_AlC_2_ power was purchased from Imnano (China). CORM-401 was purchased from MedChemExpress (China). 2-Methylimidazole (99%) and zinc nitrate hexahydrate (Zn (NO_3_)_2_·6H_2_O, 98%) were from Sigma-Aldrich (USA). Phosphate-buffered saline (PBS, pH = 7.4) was purchased from Gibco (USA). Counting Kit-8 was purchased from Abbkine (USA). SYTO 9/PI Live/Dead Bacterial Double Stain Kit was purchased from MKBio (China). Gram-negative ampicillin-resistant *Escherichia coli* (AREC) and gram-positive methicillin-resistant *Staphylococcus aureus* (MRSA) were purchased from Guangdong Detection Center of Microbiology.

### Characterizations

The shape and structure of the samples were observed by transmission electron microscopy (TEM) (JEOL, Japan) at an accelerating voltage of 80 kV. The size distribution and zeta potential of the samples were measured by a Nano ZS instrument (Malvern, UK). The structure of the samples was detected by Fourier transform infrared spectrometer (Thermo Scientific iN10, USA) and x-ray diffractometer (RIGAKU Miniflex 600, Japan). The ultraviolet (UV)-visible (vis) spectra of the samples were recorded using a UV-vis spectrophotometer (Hitachi u3900h). The surface morphology of the bacteria was characterized by a field emission biological scanning electron microscope (Hitachi SU8010, Japan).

### Preparation of monolayer Ti_3_C_2_ nanosheets (MXene)

MXene were synthesized using a previously reported chemical exfoliation method [27]. Specifically, 1.0 g of Ti_3_AlC_2_ powder was soaked in 10 ml of HF (40 wt%) and stirred at room temperature for 3 d. Following centrifugation and washing with ethanol and water, the powder was dispersed in 10 ml of dimethyl sulfoxide solution and stirred at room temperature for an additional 3 d. The collected solid was then washed several times to remove residual dimethyl sulfoxide, transferred to deionized water under a nitrogen atmosphere, and ultrasonicated for 5 h to obtain black monolayer MXene.

### Preparation of MOF (ZIF-8)

The synthesis of MOF was improved based on a previously reported experimental method. Zn (NO_3_)_3_·6H_2_O (0.149 g) was added to 10 ml of ultrapure water and then slowly poured into another 10 ml of ultrapure water containing 2-methylimidazole (0.205 g). The mixture was vigorously stirred under ice bath conditions for 45 min and centrifuged at 8,000 rpm for 10 min. It was washed 3 times with water and dried overnight under vacuum.

### Preparation of MXene@MOF (MM)

Ten milligrams of the obtained monolayer MXene nanosheets was dispersed in 10 ml of aqueous solution containing 0.149 g of Zn (NO₃)₂·6H₂O. To this solution is added 10 ml of an aqueous solution containing 0.205 g of 2-methylimidazole. The mixture is then stirred vigorously in an ice bath for 45 min, centrifuged, and washed several times with water to remove unreacted material.

### Preparation of MMC

Ten milligrams of the obtained monolayer MXene nanosheets was dispersed in a 10-ml aqueous solution containing 0.149 g of Zn (NO₃) ₂·6H₂O. Subsequently, the CORM-401 aqueous solution was added. To this solution, 10 ml of an aqueous solution containing 0.205 g of 2-methylimidazole was added. The mixture was then stirred vigorously in an ice bath for 45 min, centrifuged, and washed several times with water to remove any unreacted materials. The feed weight ratio of CORM-401 to MOF in the reaction was 1:100.

### Loading efficiency of MMC

The loading efficiency of MMC was quantified by UV-vis spectrophotometry. The MMC suspension was then centrifuged at 4 °C (10,000 rpm, 10 min) to obtain the supernatant. The concentration of free CORM-401 in the supernatant was quantified using a calibration curve based on UV absorption at 384 nm. The CORM-401 loading efficiency was calculated as follows: Loading efficiency (%) = (1 − Amount of free CORM-401/amount of added CORM-401) × 100.

### Photothermal effect of MMC

To investigate the photothermal effects of MMC nanosheets, we monitored temperature fluctuations in samples with different concentrations (0, 25, 50, 100, and 200 μg/ml) of MMC under 808-nm laser irradiation (1.5 W/cm^2^) and at different power intensities of NIR lasers. Temperature changes were recorded every minute using an infrared thermal imager. To further evaluate the photothermal stability of MMC, the suspension was subjected to an on/off cycle of NIR laser (808 nm, 1.5 W/cm^2^) irradiation for 10 min, followed by a natural cooling period of 10 min. After 4 irradiation cycles, the entire heating and cooling process was thermally imaged using an infrared thermal imager.

### Detection of reactive oxygen production

ROS production was measured using a ROS assay kit (Beyotime, China) according to the manufacturer’s instructions. Specifically, 2,7-dichlorofluorescein diacetate (DCFH-DA) was used at a dilution of 1:1,000 and added to the bacterial suspension, which was adjusted to a concentration of 1 × 10^7^ CFU/ml. The mixture was then incubated in the dark at 37 °C for 30 min. Images were acquired using a confocal laser scanning microscope (Zeiss LSM800).

### NIR-triggered CO release

The CO production of MMC was assayed by the hemoglobin (Hb) method. Briefly, 3 mg of bovine erythrocyte Hb was added to 10 ml of PBS (10 mM, pH = 7.4), and 36 mg of sodium dithionite was added under nitrogen. The solution was stirred well under nitrogen for 15 min. The samples were added and irradiated under NIR laser (808 nm, 1.5 W/cm^2^), and the absorption peaks within the 350- to 600-nm range were measured using an enzyme meter at 0, 5, 10, 15, 20, 25, and 30 min. The conversion of Hb to carboxyhemoglobin (HbCO) was quantified by the strong absorption peaks at 410 and 430 nm for HbCO and Hb, respectively. The concentration of CO released (Cco) was calculated using the following equation:

$$\begin{array}{c} C_{\mathrm{co}}=C_{\mathrm{Hb}}\times \left(528.6\times I_{410\text{nm}-}\;304\times I_{430\text{nm}}\right)/\\ \;\left(216.5\times I_{410\text{nm}}+442.4\times I_{430\text{nm}}\right) \end{array}$$

C_co_ is the released CO concentration, C_^Hb^_ is the initial Hb concentration, and *I*_410nm_ and *I*_430nm_ are the UV absorptions at 410 and 430 nm of the above mixed solutions.

### Bacterial strains and cell culture

AREC and MRSA were incubated on Luria-Bertani (LB) agar plates at 37 °C for 16 h, and single colonies were isolated and purified. Purified single colonies were then picked with an inoculation loop, inoculated into LB broth, and incubated on a shaker (37 °C, 200 rpm) until logarithmic growth phase. After centrifugation to wash the bacteria, the bacteria were resuspended in 0.9% saline for subsequent experiments. Mouse skin fibroblasts (L929) and human oral keratinocytes (HOKs) were cultured in minimum essential medium and Dulbecco's modified Eagle medium supplemented with 10% fetal bovine serum and 1% antibiotics at 37 °C, 5% CO_^2^_, respectively.

### In vitro antimicrobial assay

The in vitro antimicrobial properties of MMC were evaluated using plate count and growth curve methods. MRSA (gram-positive bacteria) and AREC (gram-negative bacteria) were diluted to 1.0 × 10^6^ CFU/ml and incubated with different concentrations (0, 25, 50, 100, and 200 μg/ml) of MMC and MMC combined with NIR into sterile 96-well plates in a total volume of 200 μl. The mixtures were incubated at 37 °C for 24 h. Postincubation, the absorbance of the mixture was determined at 600 nm. The NIR-treated group was irradiated under NIR laser for 10 min. The minimum inhibitory concentration was defined as the lowest drug concentration that inhibited bacterial growth, maintaining the initial optical density (OD). The minimum bactericidal concentration was determined using the dilution plate method and was defined as the lowest drug concentration that killed the bacteria.

The bacterial suspension of 1.0 × 10^6^ CFU/ml was treated with different experimental groups (PBS, PBS +NIR, MXene, MXene +NIR, MM, MM +NIR, MMC and MMC +NIR) by minimum bactericidal concentration, incubated for 2 h and then coated on agar solid medium, and finally photographed and counted after incubation for 16 h at 37 °C. Additionally, bacterial growth curves under different experimental treatments were determined. Test tubes containing fresh LB medium (pH 5.5) were inoculated with a suspension of 1.0 × 10^6^ CFU/ml and incubated with different experimental groups of the same concentration for 20 h on a constant temperature shaker (37 °C, 200 rpm), and the absorbance at 600 nm was measured by a UV-vis spectrophotometer at 2-h intervals.

### Bacterial live/dead assays

Live/dead detection of bacteria was performed using SYTO 9/PI Live/Dead Bacterial Double Stain Kit. A bacterial suspension with a 1.0 × 10^8^ CFU/ml was treated with PBS group, MXene group, MM group, and MMC group under laser or no laser condition. The bacteria were collected after 2 h of incubation and costained with SYTO 9 green fluorescent nucleic acid stain and propidium iodide (PI) fluorescent dyes under dark conditions for 15 min, excess dye was removed by washing with PBS, and live and dead bacterial cells were observed by confocal laser scanning microscopy. Finally, fluorescence images were quantitatively analyzed using ImageJ.

### Scanning electron microscope morphology observation of bacteria

After treatment, various bacteria were collected, centrifuged, rinsed several times with PBS, and preserved in glutaraldehyde (2.5%) overnight. A gradient dehydration process was performed using ethanol concentrations of 30%, 50%, 70%, 90%, and 100%. The dehydrated bacterial suspension was then dispensed onto a silicon wafer, air dried, coated with gold, and examined under a scanning electron microscope to observe the surface morphology of the bacteria.

### Crystalline violet staining for determination of biofilm biomass

For the construction of bacterial biofilm, a bacterial suspension at a concentration of 1 × 10^8^ CFU/ml was added to 48-well plates and incubated at 37 °C for 48 h. The medium was replaced with fresh medium every 24 h. The bacterial biofilm was then incubated at 37 °C for 48 h, and the medium was replaced with fresh medium every 24 h. Finally, the wells were rinsed 3 times with sterile PBS buffer to remove planktonic bacteria. The bacterial biofilm was then fixed with methanol for 15 min, the methanol was aspirated, allowed to air dry, and then stained with 0.1% crystal violet solution for 20 min. The wells were then carefully washed 3 times with PBS to remove excess dye and air dried for 1 h. After drying, anhydrous ethanol was added to the wells, and the absorbance of the remaining crystal violet was measured at 590 nm using a 96-well plate reader to determine biofilm biomass. Biofilm eradication and inhibition rates were calculated as: Biofilm eradication/inhibition rate (%) = (1 − OD _test_ / OD _control_) × 100%.

### Characterizations of biofilm morphology by CLSM

MRSA and AREC bacterial suspensions (1 ml at 10^7^ CFU/ml) were dispensed into the wells of 48-well plates and incubated at 37 °C for 48 h to promote the formation of bacterial biofilms. Following the corresponding treatments, all samples were fixed with absolute alcohol for 15 min, stained for 30 min, and washed with PBS to remove excess dye. Finally, CLSM was used to observe the morphology of the bacterial biofilms stained with SYTO 9.

### In vitro cytotoxicity assay

Toxicity evaluation of L929, HOK, human umbilical vein endothelial cells (HUVECs), and RAW264.7 cells was performed using the cell counting kit-8 (CCK-8) method. L929, HOK, HUVECs, and RAW264.7 cells were seeded into 96-well plates (2 × 10^3^ cells per well) and incubated at 37 °C for 24 h to make the cells adhere. Subsequently, each well was incubated with 100 μg/ml MXene, MM, MMC, or different concentrations (0, 25, 50, 100, 200, and 300 μg/ml) of MMC. After 1, 2, and 3 d of incubation, respectively, each well was incubated with CCK-8 solution for 4 h. The absorbance of the samples at 490 nm was measured using an enzyme meter. Cytotoxicity was expressed as cell viability in 3 replicate determinations, calculated as: cell viability (%) = OD_t_/OD_n_ × 100% (OD_t_: absorbance of experimental group, OD_n_: absorbance of control group).

### Wound healing scratch assays

HUVECs and L929 cells were seeded onto 6-well plates at a density of 1 × 10^6^ cells per well and cultured in a 37 °C, 5% CO_2_ incubator. Once the cells reached approximately 90% confluence, a 1-mm-wide gap was created in the middle of the well using a pipette tip. The cells were then washed with PBS and treated with serum-free medium containing the materials according to the experimental groups. Images were randomly captured using a light microscope (Olympus, Japan) at 24 h posttreatment (magnification, ×100). The width of each gap was statistically measured using ImageJ software, and the cell migration rate was calculated using the formula: Migration rate (%) = (*A*_0_ − *A*_t_) / *A*_0_ × 100%, where *A*_0_ represents the initial scratch area, and *A*_t_ represents the area at 24 h postscratch.

### Real-time quantitative polymerase chain reaction (RT-qPCR)

HUVECs and RAW264.7 cells were cocultured with MXene, MM, and MMC in 6-well plates at specific time points. Total RNA was extracted using the EZ-press RNA Purification Kit (EZBioscience, USA) following the manufacturer’s instructions. RNA (500 ng) was reverse transcribed to cDNA as described elsewhere, using glyceraldehyde-3-phosphate dehydrogenase (GAPDH) as an internal control. All primer sequences are listed in Table. The RT-qPCR conditions were set as follows: denaturation at 95 °C (30 s), 40 cycles at 95 °C (5 s), and 60 °C (30 s, annealing), followed by a final dissociation step. The RT-qPCRs were performed using the SYBR Green Pro Taq HS Mix (Accurate Biotechnology, China) and the Agilent RTqPCR System (Agilent, USA). The reactions were measured in triplicate.

**Table.** The primers of the detected genes

Name	Primer	
TNF-α	F: 5’CGGGCAGGTCTACTTTGGAG-3’	R: 5’ACCCTGAGCCATAATCCCCT-3’
IL-6	F: 5’CTGCAAGAGACTTCCATCCAG-3’	R: 5’AGTGGTATAGACAGGTCTGTTGG-3’
VEGF	F: 5’ AGGCTCCAGGGCATTAGACA-3’	R: 5’ GCGGATCAAACCTCACCAAG-3’
CD31	F: 5’AACAGTGTTGACATGAAGAGCC-3’	R: 5’TGTAAAACAGCACGTCATCCTT-3’
GAPDH	F: 5’GCACCGTCAAGGCTGAGAAC-3’	R: 5’TGGTGAAGACGCCAGTGGA-3’

### Hemolysis assays

The hemocompatibility of MMC was evaluated by a hemolysis assay. We collected fresh blood from mice and rinsed it once with PBS. We then diluted the rinsed blood by mixing it with PBS in a ratio of 1:1.25. This diluted blood was combined with different concentrations of MMC (ranging from 0 to 300 μg/ml) and incubated at 37 °C for 4 h, followed by centrifugation at 4 °C for 3 min at 5,000 rpm. The supernatant from each mixture was transferred to a 96-well plate, and we measured its absorbance at 545 nm using an enzyme meter to determine the rate of hemolysis. For our controls, we used ultrapure water as the positive control and PBS as the negative control. The hemolysis rate (%) was calculated using the formula: [(A_t_ − A_n_)/(A_p_ − A_n_)] × 100%, where A_t_ is the absorbance of the experimental group, A_p_ is the absorbance of the positive control group, and A_n_ is the absorbance of the negative control group.

### In vivo accessment of wound infection healing

Female db/db mice (6 to 8 wk old) were purchased from Changzhou Cavins Laboratory Animal Co., Ltd. All animal experiments were performed in accordance with the ethical guidelines for the care and use of laboratory animals and were approved by the Animal Ethics Committee of Guangdong Huawei Testing Co., Ltd. (HWT-BG-117b; NO:202311003). They were maintained at a controlled ambient temperature of 24 ± 1 °C and a relative humidity of 50% to 70%. General anesthesia in mice was induced by an intramuscular injection of anesthetics (1 ml/kg), including Zoletil 50 (2 mg/kg; Virbac, China), Sumianxin-II (0.02 ml/kg; Sheng xin, China), and sterilized saline. The mice were positioned supine on a 37 °C heating pad, and the dorsal region of each mouse was shaved and sterilized. The housing method for each mouse has been adopted as one-per-cage, with clear cage numbering and double ear tagging implemented to enhance the recognizability of the experimental subjects. The selection of samples in the experiment has been ensured to be random, thereby guaranteeing the objectivity and reliability of the results. A circular, full-thickness wound 8 mm in diameter was created on the back of the mouse. The wounds were then infected with an MRSA bacterial suspension (1 × 10^8^ CFU/ml, 20 μl). After 24 h, the wounds were treated with PBS, MXene, MM, and MMC, followed by NIR irradiation (1.5 W/cm^2^) for 10 min. Body weights of the mice were recorded daily. Wound progression was photographed at specified intervals, and the wound healing rate (%) was calculated using the formula: (original surface area − current surface area)/original surface area × 100%.

After 14 d, the mice were euthanized. All skin tissues, hearts, livers, spleens, lungs, and kidneys were collected and subjected to histologic examination by hematoxylin and eosin (H&E) and Masson’s staining. Serum samples from the treated diabetic mice were collected for biochemical analyses to evaluate liver and kidney functions by various indices. Skin tissue bacteria were cultured on solid medium and incubated at 37 °C for 16 h. In vivo bacterial viability was determined by colony counting.

### Histological and immunohistochemical evaluation

After 14 d, the mice were euthanized, and all wound tissues as well as the heart, liver, spleen, lungs, and kidneys were excised. The collected samples were fixed in 4% paraformaldehyde, dehydrated, paraffin-embedded, and sectioned. H&E staining was performed on the vital organs (heart, liver, spleen, lungs, and kidneys) to assess the in vivo toxicity of the drug. The healing of wound tissues and collagen deposition were evaluated using H&E staining and Masson’s trichrome staining. The expression of interleukin-6 (IL-6) and vascular endothelial growth factor (VEGF) in the wounds was detected by immunohistochemical methods, with quantitative analysis performed using ImageJ software.

### Statistical analysis

Statistically significant differences between treatment groups were determined by 2-tailed Student *t* test. Ordinary 2-way analysis of variance was used to analyze differences among groups, followed by Tukey’s multiple comparisons test. Differences between different groups at *P < 0.05 was considered as statistically significant. All data represent mean ± SD of data from at least 3 independent experiments.

## Results

### Synthesis and characterization of MMCs

**Fig. 1.**
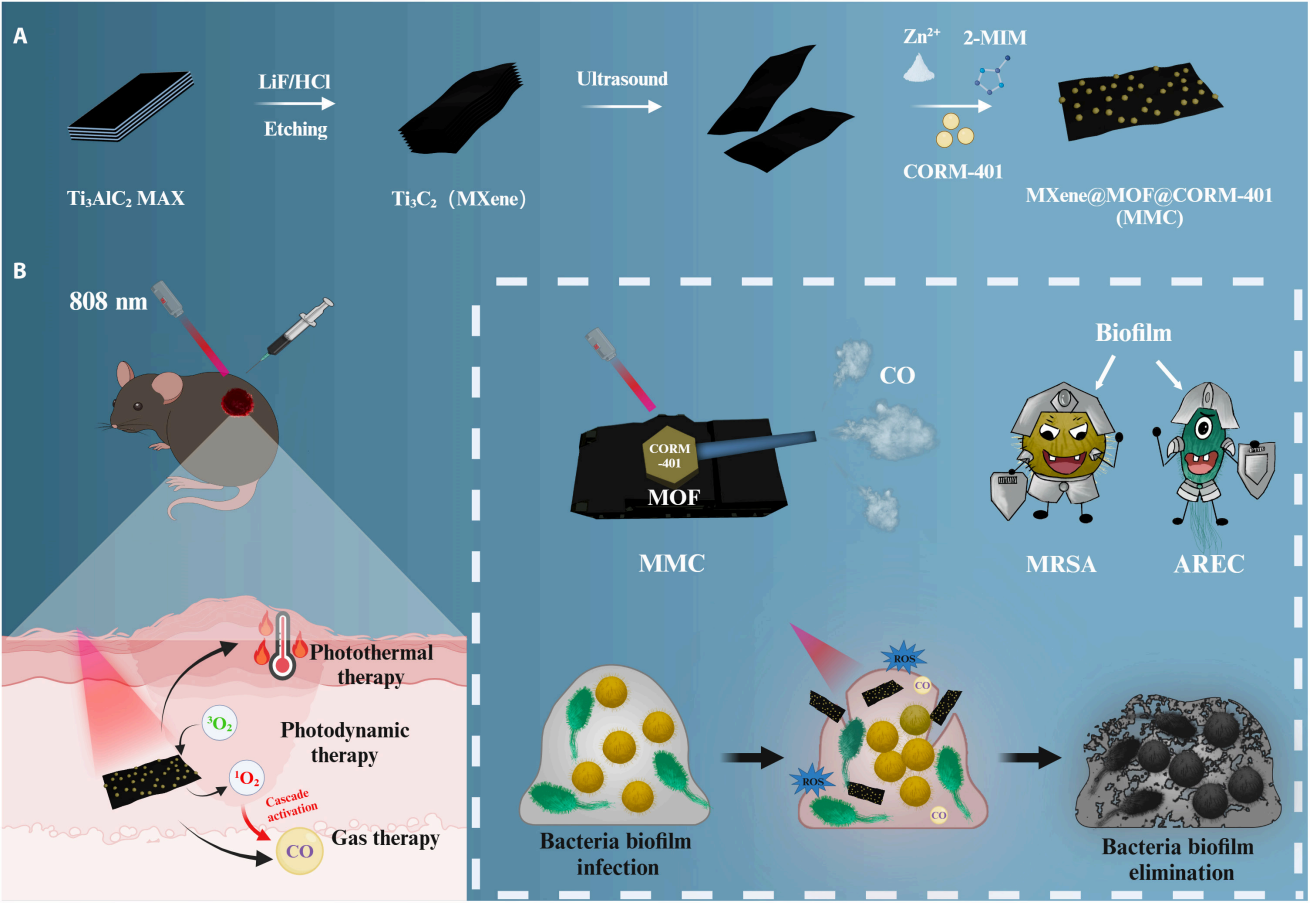
Synthesis, potential antibacterial mechanism, and biomedical application of MMC. (A) The schematic diagram illustrating the synthesis process of MMC. (B) The synergistic treatment of MMC with triple modes of photothermal/photodynamic/gas therapy multidrug-resistant bacterial infections.

Nanosheet MMCs were synthesized by encapsulating CORM-401 in MOF via the one-pot method. The synthesis process was basically the same as that shown in Fig. 1. The key to the one-pot synthesis of MMC lies in precisely controlling the reaction conditions to ensure the uniform binding between MXene and MOF, as well as the effective encapsulation of CORM-401. The negative charges on the MXene surface are attributed to the negatively charged groups (e.g., −F, −OH, and other groups) that carry negative charges [31,32]. These negatively charged groups provide active attachment sites for the surface functionalization of MXene. In order to grow the MOFs in situ on the MXene surface, zinc salt was first mixed with an aqueous solution of MXene. The positively charged zinc ions could then bind with the negatively charged functional groups on the MXene surface by electrostatic adsorption, and the zinc ions combined with ligands to form a uniformly distributed MOF on the MXene surface. We reduced the temperature of the synthesis environment by using an ice bath to slow down the crystal growth rate, which allowed the formation of smaller and more uniformly sized ZIF-8 nanoparticles formed on the MXene surface.

**Fig. 2.**
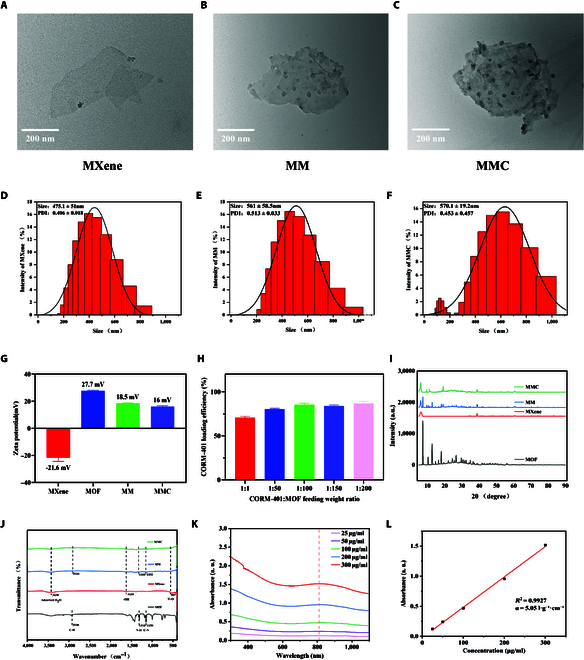
Characterization of MMC. (A to C) TEM image of MXene, MM, and MMC. (D to F) The size distribution of MXene, MM, and MMC. (G) Zeta potential of MXene, MOF, MM, and MMC. (H) Loading efficiency of CORM-401-MOF at different ratio. Data are mean ± SD (*n* = 3). (I) XRD diagram of MXene, MOF, MM, and MMC. (J) Fourier transform infrared spectra of MXene, MOF, MM, and MMC. (K) Absorption spectra of MMC with various concentrations (25 to 300 μg/ml). (L) Plot of absorbance changes at 808-nm wavelength versus different concentrations of MMC.

TEM revealed that MXene, MM, and MMC presented a lamellar structure with sharp edges (Fig. 2A to C), with average diameters of 475.1 ± 51 nm, 561 ± 58.5 nm, and 570.1 ± 19.2 nm, respectively (Fig. 2D to F). We assessed the stability and dispersibility of the material in a physiological environment. Digital images showed no noticeable change in the MMC colloidal dispersion after being incubated in PBS and water for 24 h, exhibiting a deep gray color. The dispersibility of the MMC colloid was studied using a red laser beam, where the incident light was scattered by the colloidal nanosheets (Tyndall effect) (Fig. S1). This indicates that MMC has excellent stability and dispersibility in a physiological environment. MOF particles on MM and MMC were uniformly distributed and of similar size. Zeta potentials showed that MXene, MOF, MM, and MMC had potentials of −21.6, 27.7, 18.5, and 16 mV, respectively (Fig. 2G).

During the one-pot synthesis process, the timing of CORM-401 introduction has a important impact on the loading efficiency. CORM-401 is introduced during the early growth phase of the MOF, allowing the drug to be captured and encapsulated within the internal pores of the newly formed MOF crystal structure. On the one hand, this can achieve a high loading efficiency, and on the other hand, the encapsulation of CORM-401 within the MOF can reduce direct contact with the external environment, thereby enhancing the structural and chemical stability of MMC. To determine the loading efficiency of CORM-401 on MOF, we measured different mass ratios (1:1, 1:50, 1:100, 1:150, and 1:200) of CORM-401 to MOF. To determine the loading efficiency of different concentrations of MOF materials and CORM-401, it was found that the loading efficiency reached more than 80% when the mass ratio of CORM-401 to MOF was 1:100 (Fig. 2H). Considering both loading efficiency and the concentration of CORM-401, we selected MMC with a mass ratio of 1:100 for further study. The successful synthesis of MMC was further confirmed by x-ray diffraction (XRD) and Fourier transform infrared spectroscopy. XRD analysis showed that the profiles of MXene and MOF were consistent with previously reported standard profiles. The characteristic peaks of MXene and MOF in MM indicated successful synthesis. The slight shift in the characteristic peaks of MMC compared to MM was likely due to the interaction of CORM-401 with the crystal structure, affecting the diffraction peak positions (Fig. 2I). Fourier transform infrared spectroscopy identified functional groups in MXene, MOF, MM, and MMC (Fig. 2J). In MMC, distinct characteristic absorption peaks appear at 557, 1,630, and 3,430 cm^−1^, attributed to the bending vibrations of Ti–O, –OH, and H_2_O on MXene. Multiple peaks at 1,150, 1,310, and 2,930 cm^−1^ are attributed to the stretching vibrations of C-N, N-H, and C-H bonds in MOF. Compared to the infrared spectra of MM and MMC, there is no significant difference, proving that CORM-401 piggybacking occurs through physical adsorption without chemical bond formation. The UV-visible absorption spectra of MMC at different concentrations (25, 50, 100, 200, and 300 μg/ml) are shown in Fig. 2K and L. MMC exhibits strong absorption in the NIR spectral range (700 to 900 nm) with a considerable mass extinction coefficient of 5.05 l·g^−1^·cm^−1^. These results confirm the successful synthesis of MMC nanostructures.

### Photothermal properties of MMC

**Fig. 3.**
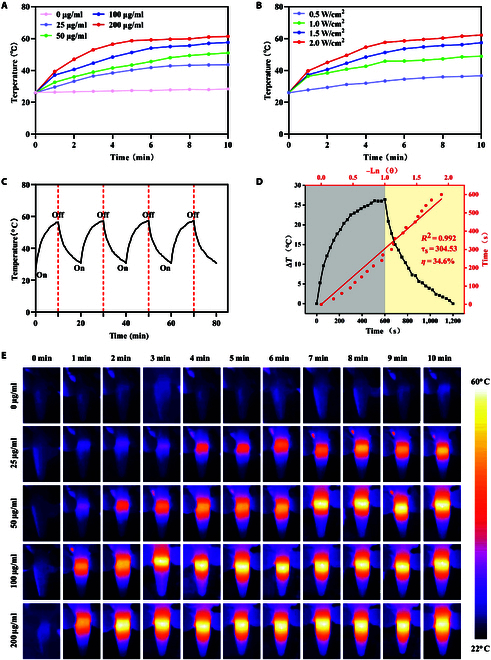
Photothermal properties of MMC. (A) Concentration-dependent photothermal heating curves of MMC (0, 25, 50, 100, and 200 μg/ml) irradiated by 808-nm laser (1.5 W/cm^2^, 10 min). (B) Photothermal heating curves of MMC (100 μg/ml) irradiated by 808-nm laser (0.5 to 2.0 W/cm^2^, 10 min). (C) Recycling–heating profiles of MMC dispersions (100 μg/ml) for 4 on/off cycles. (D) Temperature changes of MMC dispersions (100 μg/ml) under irradiation with 808-nm laser (1.5 W/cm^2^, 10 min). Linear time data versus −ln θ obtained from the cooling period. (E) Thermal images of MMC solution (at different concentrations) irradiated with an 808-nm laser (1.5 W/cm^2^, 10 min) (mean ± standard error of the mean [SEM], *n* = 3).

PTT is a therapeutic method that uses a photothermal agent to convert light energy into heat energy under the irradiation of an external light source, such as NIR laser. MXene exhibits a unique absorption characteristic in the NIR range, which enables MXene to generate an excellent photothermal effect when irradiated with NIR light at a wavelength of 808 nm. Figure 3A shows the temperature change of MMC nanosheet suspension at different concentrations (0 to 200 μg/ml). Under the irradiation of NIR (808 nm, 1.5 W/cm^2^), the final temperatures of MMC suspensions at different concentrations increased by 16.7, 23.4, 28.7, and 34.9 °C, respectively, while the temperature change of water was negligible. Among them, the above trend was also confirmed in the infrared thermograms at different time points (Fig. 3E). In addition, the MMC nanosheet suspensions exhibited better photothermal conversion with increasing power of NIR laser (Fig. 3B). The MMC nanosheets exhibited power density-dependent (0.5 to 2.0 W/cm^2^) and concentration-dependent (0 to 200 μg/ml) photothermal conversion. To evaluate the photothermal stability and reproducibility of the MMC nanosheet suspensions, 4 on/off cycles of irradiation were performed under NIR irradiation (808 nm, 1.5 W/cm^2^), and no noticeable temperature changes were observed during the cyclic heating process, indicating that the MMCs had excellent cyclic stability (Fig. 3C). Meanwhile, one of the cycles was randomly selected, and the photothermal conversion efficiency of MMC was calculated to be 34.6% (Fig. 3D). As shown in the summary, the excellent photothermal conversion ability and photothermal stability of MMC nanosheets were mainly attributed to the large specific surface area and efficient photothermal conversion ability of MXene nanosheets.

### Photodynamic properties and mechanisms of MMC

**Fig. 4.**
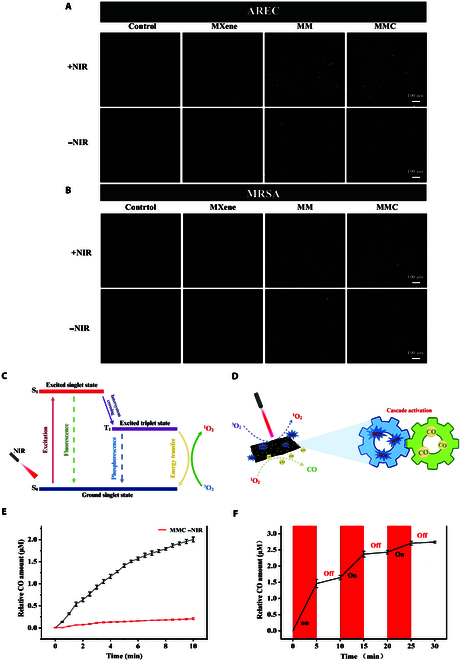
Photodynamic properties and CO release of MMC. (A and B) Confocal fluorescence images of AREC and MRSA stained with DCFH-DA in different treatments. Scale bar: 100 μm. (C) Simplified diagram of singlet oxygen generation in PDT. (D) CO release mechanism through cascade events of photoinduced ^1^O_2_ generation and ROS-activated CO molecule dissociation. (E) Concentration variation of the released CO during NIR irradiation (1.5 W/cm^2^) for 10 min (MMC: 100 μg/ml). (F) CO release profile of MMC (100 μg/ml) without or with laser irradiation conditions.

We used DCFH-DA fluorescent probe to detect intracellular ROS levels in AREC and MRSA (Fig. 4A and B). Specifically, the control and MXene groups showed extremely low levels of ROS generation in both AREC and MRSA, whereas the MM group and the MMC group showed a increase in intracellular ROS levels after laser irradiation (Fig. S2). Notably, a small amount of ROS was also detected in the MM −NIR group and the MMC −NIR group, which we speculate may be due to the indirect generation of a small amount of free radicals and ROS through disruption of bacterial cell membranes or reaction with bacterial proteins, etc. In contrast, studies have shown that the zinc-based MOF reacts with phosphate and sulfur radicals in the bacterial membrane proteins and produces ROS [33]. In addition to this, zinc oxide also possesses excellent catalytic properties. Upon contact with bacteria, it can induce oxidative stress, leading to the production of ROS and free radicals, thereby exerting a potent antimicrobial effect [34].

PDT is a technology in which a photosensitizer reacts photochemically with molecular oxygen after light excitation at a specific wavelength to produce ROS to achieve antibacterial and anticancer effects, among others. A small amount of ROS is present in bacteria/cells, and endogenous ROS are involved in respiration, metabolism, and physiological regulation [35]. Excessive ROS production leads to an imbalance in intracellular redox homeostasis, resulting in physiological dysfunction and bacterial death. The principle of NIR-activated MMC nanosheets for PDT is shown in Fig. 4C. In PDT, upon photoexcitation, the electrons from the ground state (S_0_) of PS are converted to a single wire excited state (S_1_). Some of the absorbed energy is returned to the ground state in the form of fluorescence production, and some is released through intersystem crossing, which facilitates the movement of electrons to the triplet excited state (T_1_). The triplet excited state electrons undergo 2 types of reactions [32]. Type I uses the electrons to react with triplet oxygen (^3^O_2_) or water to produce hydrogen peroxide (H_2_O_2_), superoxide anion (O_2_·^−^), hydroxyl radical (–OH), etc., while Type II is a triplet electron directly transferring energy to ^3^O_2_ in the periphery, which is converted to singlet oxygen (^1^O_2_). The active site region on the surface of photocatalytic materials is able to trap O_2_ and H_2_O molecules, which can generate a sufficient amount of ROS after catalytic oxidation, and this is the main source of ROS generation in the antimicrobial process of photocatalytic materials [36]. As an emerging class of photocatalytic materials, MOFs have huge advantages in photocatalysis due to their unique properties, such as their porous structure, high crystallinity, and designability [37]. Studies have shown that MOFs act in PDT mainly by Type II energy transfer, which not only has a long action period and is drug-free but also the Type II energy transfer process is much faster than that of Type I electron transfer [38,39].

### CO gas release performance of activated oxygen triggered MMC

CORM-401 is an oxidant-sensitive CO-releasing molecule, and CORM-401 rapidly releases CO upon stimulation with ROS, thus predicting a cascade reaction in which CO gas molecules are activated to be released from MMCs by ^1^O_2_ generated by photoinduction (Fig. 4D). To confirm our prediction, the Hb method was used to detect CO release from MMC. CO production was quantified by measuring the conversion of hemoglobin to HbCO using the strong absorption peaks of HbCO and Hb at 410 and 430 nm, respectively. The CO gas release behavior induced by ROS was investigated by irradiating the MMC-loaded mixed solution with lasers (808 nm, 1.5 W/cm^2^). The results showed a rapid release of CO gas under NIR irradiation, with a rapid release of 2.0 μM CO gas after 10 min, while a small amount of CO was released slowly without laser irradiation (Fig. 4E). Interestingly, cyclic irradiation with 3 cycles of NIR can further respond to the controlled release of CO (Fig. 4F). Under NIR irradiation, the MMC nanosheets rapidly released 1.46 μM CO gas within 5 min, while the release of CO was negligible after the laser was turned off. In conclusion, a cascade response to CO gas release was achieved by photoinduction-generated ROS, and this controlled gas therapy has an important role in antimicrobial therapy.

### In vitro antibacterial activity of MMC

**Fig. 5.**
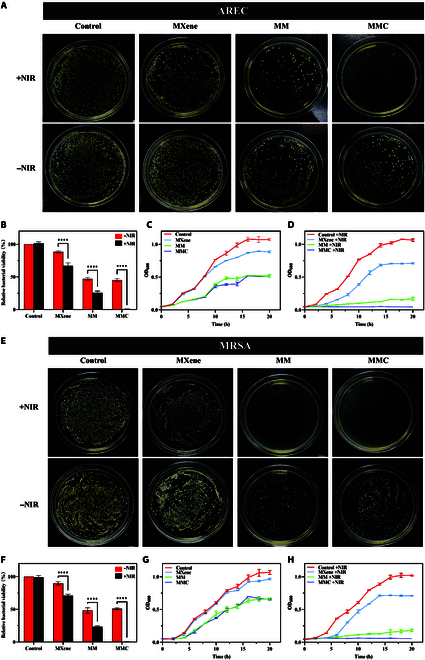
Antibacterial activity of MMC in vitro. (A, B, E, and F) Agar plate photographs and the relative bacterial survival. Agar plate photographs of bacterial colonies formed by (B) AREC and (F) MRSA after upon the indicated treatments. The summaries for relative bacterial viability in (B) AREC and (F) MRSA are for data from (A) and (E), respectively. (C and D) The growth curve of AREC incubated with MXene, MM, and MMC (100 μg/ml) for 20 h. (G and H) The growth curve of MRSA incubated with MXene, MM, and MMC (100 μg/ml) for 20 h (mean ± SEM, *n* =3, *P < 0.05, **P < 0.01, ***P < 0.001). OD_600_, optical density at 600 nm.

To evaluate the antibacterial effect of MMC on gram-negative and gram-positive bacteria, we performed plate counts of AREC and MRSA (Fig. 5A, B, E, and F). We found that all untreated bacteria survived before and after laser irradiation, confirming that laser irradiation alone had a negligible effect on AREC and MRSA. When bacteria interact with MXene nanosheets, the survival rate of the bacteria is only slightly reduced due to the sharp edges of MXene nanosheets that can damage the surface membrane structure of the bacteria. In addition, MXene can further kill bacteria by photothermal action under laser irradiation [40,41]. The survival rates of AREC and MRSA were notably decreased in the MM group, the MM +NIR group, the MMC group, and the MMC +NIR group. In the NIR-treated MMC group, both strains showed little or no survival on the plates. In addition, the bacterial growth curves by measuring the absorbance (optical density [OD] = 600 nm) further confirmed that the treated group had some antibacterial effect without irradiation, whereas the bacteria in the laser-irradiated MMC group were almost completely killed (Fig. 5C, D, G, and H).

**Fig. 6.**
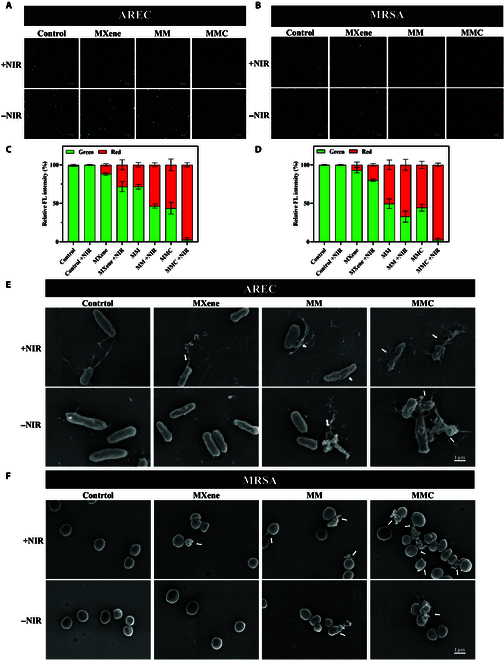
Morphological changes in bacteria after incubation with MMC. (A to D) Fluorescence staining images of (A) AREC and (B) MRSA after the indicated treatments. Live and dead bacteria were stained (green fluorescence) using SYTO 9; additionally, dead bacteria were stained (red fluorescence) by PI. Scale bar: 100 μm. The corresponding relative fluorescence intensity in (C) AREC and (D) MRSA. (E and F) Scanning electron microscope images of AREC and MRSA in different treatments. White arrows indicate the membrane rupture of the bacteria. Scale bar: 1 μm.

The antibacterial efficacy of MMC nanosheets was further validated using SYTO 9/PI dual fluorescence staining (Fig. 6A to D). These experiments showed that all treatment groups exhibited effective bactericidal activity, and the synergistic photothermal/photodynamic/gas therapy under MMC +NIR had the strongest antimicrobial activity against AREC and MRSA.

To further investigate the antibacterial mechanism of the MMC +NIR antibacterial system, we observed changes in the surface morphology of bacteria in different groups using scanning electron microscopy (Fig. 6E and F). AREC and MRSA in the control group exhibited typical rod and spherical morphologies with intact cell membranes, whereas bacterial surfaces treated in the MXene group displayed slight wrinkles and distortions, indicating minor defects in the integrity of the bacterial cell membranes. The bacterial cell membranes in the MM group and the MM +NIR group exhibited further irregularities and disruptions, while those in the MMC +NIR group showed the most severe morphological damage, with nearly complete loss of integrity.

### Elimination of the established biofilm

**Fig. 7.**
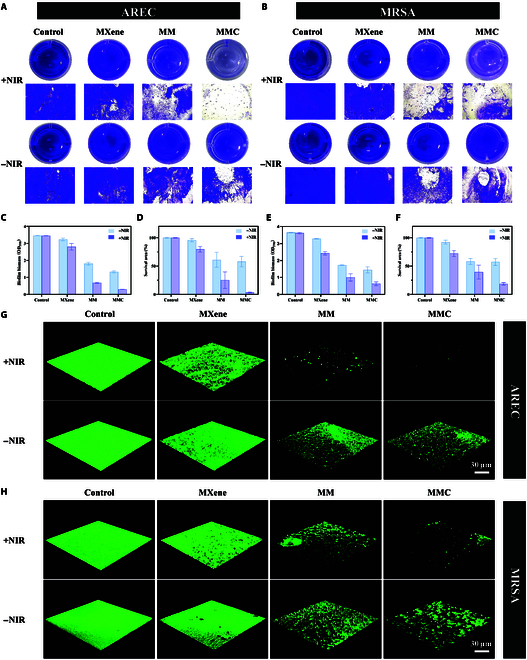
Elimination of the established biofilm. (A and B) Crystal violet staining of the biofilm after different treatments. (C) Statistical analysis of the relative biomass of the AREC biofilm. (E) Statistical analysis of the relative biomass of the MRSA biofilm. (D and F) Survival area of AREC and MRSA biofilms after different treatments. (G and H) SYTO 9 staining of the biofilm after different treatments.

Bacterial biofilms are complex structures formed by bacteria that adhere to biotic or abiotic surfaces and secrete extracellular polymers, an extracellular matrix that protects the bacteria within the biofilm. Biofilms protect microorganisms from the host immune system and confer resistance to antimicrobial drugs such as antibiotics [42]. Once biofilms are formed, bacteria can adapt to the host microenvironment, causing inflammation and leading to persistent infections. Removing formed biofilms is usually more difficult than killing planktonic bacteria [43]. Therefore, we evaluated the efficacy of MMC in eradicating established biofilms using crystal violet staining, with AREC and MRSA biofilms as models (Fig. 7A to F). NIR irradiation alone had no effect on the biomass of biofilms in the PBS group. The biomass of MXene-treated biofilms decreased slightly compared to the untreated group, indicating weaker biofilm resistance. For AREC biofilm, the MM +NIR and MMC +NIR groups eradicated 74.8% and 96.4% of the biofilm, respectively. For MRSA biofilm, the MM +NIR and MMC +NIR groups eradicated 60.6% and 81.4% of the biofilm, respectively. This not only indicates that the 3-modality synergistic treatment of PTT, PDT, and gas therapy caused the greatest damage to the biofilm but also confirms that CO, a small-molecule gas, can penetrate into the biofilm to exert its effects without being hindered by extracellular polymers. In addition, CLSM showed similar results (Fig. 7G and H). The control AREC and MRSA biofilms were intact and showed strong green fluorescence, whereas the MXene +NIR-treated biofilms were limited by high temperature. The MM +NIR-treated group showed further enhancement in antibiofilm ability due to PDT and Zn^2+^ release, while the MMC group exhibited the strongest antibiofilm ability, and the least green fluorescence was detected. All these results indicated that MMC could achieve good bacterial biofilm eradication through PTT, PDT, and CO induced by ROS. It is worth noting that although the MMC group showed good biofilm removal for both bacteria, the AREC biofilm appeared more sensitive to MM and MMC than MRSA. However, there are no clear studies demonstrating the ease of removal of MRSA biofilm compared to AREC biofilm. In fact, biofilm removal can be affected by various factors, such as the thickness of the biofilm, the environment in which it is located, and the method of removal [44,45]. Therefore, the reasons for differences in the removal efficacy of temperature, ROS, and CO gas for AREC and MRSA biofilms need further investigation in the future.

### Biological performance of MMC

**Fig. 8.**
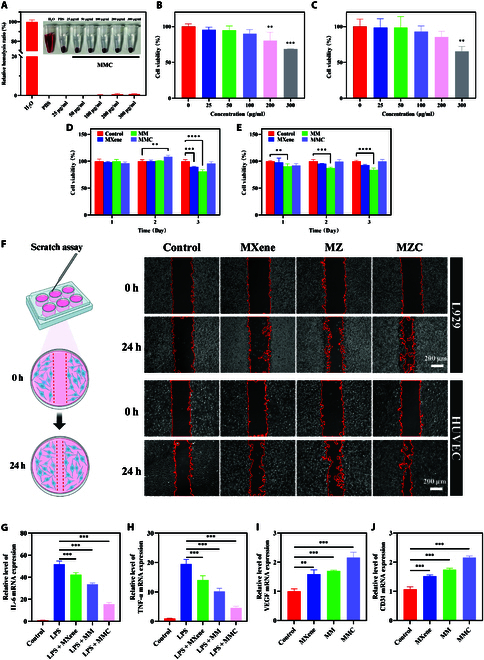
Biological performance of MMC. (A) In the hemolysis assay, erythrocytes were incubated with different concentrations (25, 50, 100, 200, and 300 μg/ml) of MMC, and PBS and deionized water were used as negative and positive controls, respectively, to ensure the accuracy and reliability of the results. The supernatant of the MMC-treated group was clear, and even at a high concentration (300 μg/ml), the hemolysis rate of MMC was only 0.74%, indicating good blood biocompatibility. This is well below the clinical requirement for the hemolysis rate of biomaterials (<5%). (B and C) Cell viability of L929 and HOK cells after treatment with MXene, MM, and MMC (100 μg/ml) for 3 d. (D and E) Cell viability of L929 and HOK cells after treatment with different concentrations of MMC for 3 d. (F) Scratch assay after 24 h of incubation. Scale bar: 200 μm. (G) IL-6 gene expression level of RAW264.7 cells treated with lipopolysaccharide (LPS), LPS + MXene, LPS + MM, or LPS + MMC. (H) TNF-α gene expression level of RAW264.7 cells treated with LPS, LPS + MXene, LPS + MM, or LPS + MMC. (I) VEGF gene expression level of RAW264.7 cells treated with LPS, LPS + MXene, LPS + MM, or LPS + MMC. (J) CD31 gene expression level of RAW264.7 cells treated with LPS, LPS + MXene, LPS + MM, or LPS + MMC.

Cell migration capacity is an essential indicator of wound healing. We further explored the in vitro cell migration behavior of MMC through scratch assays, assessing the cell migration effect by calculating the rate of reduction in scratch size. We used HUVECs and L929 cells, which have high migration capabilities, as models for the scratch assays. The results showed that the MXene group, MM group, and MMC group all promoted cell migration (Fig. 8F). Among them, the MMC group exhibited the best migration effect, with the migration rate of L929 and HUVECs reaching 43.78% and 59.25%, respectively (Fig. S3). The above results indicate that MMC can promote the migration of L929 and HUVECs to the wound area during the healing process, which is beneficial for skin tissue regeneration.

During the wound healing process, the dynamic interaction of bacterial infection, inflammatory response, and angiogenesis constitutes the key to tissue repair. After bacteria invade the wound, they and their toxins will cause an immune response in the body, leading to inflammation. At the same time, newly formed blood vessels provide the necessary nutrients for the wound, which is an essential part of tissue regeneration and repair. We delved into the potential impact of “nanotanks” in this complex process. As shown in Fig. 8G and H, both MM and MMC effectively reduced the mRNA levels of inflammatory markers IL-6 and tumor necrosis factor-α (TNF-α). Platelet-endothelial cell adhesion molecule (CD31) is a marker of vascular endothelial cells that can reflect the state of endothelial cells, and VEGF is the main factor promoting the formation of new blood vessels, playing a key role in angiogenesis. PCR was used to detect the expression of VEGF and CD31 in HUVECs in different treatment groups (Fig. 8I and J). The results showed that the mRNA expression levels of VEGF and CD31 in the drug groups were upregulated, especially in the MMC group, which was the most obvious. The MXene and MM groups showed little difference in the expression levels of VEGF and CD31. It is worth noting that CO, as a key molecule released by the “nanotank”, has shown extraordinary versatility in wound healing, including antimicrobial and anti-inflammatory properties, as well as a positive effect on angiogenesis. In addition, an appropriate amount of ROS can promote cell proliferation, differentiation, and migration, while an excessive amount of ROS may cause oxidative stress damage and inhibit tissue repair. Studies have found that the microenvironment of diabetic wounds will promote premature aging of cells, including various key cells such as endothelial cells, fibroblasts, and mesenchymal stem cells, making it difficult for senescent cells to play a role in wound healing [46]. Senescent cells have been proven to drive inflammatory responses, and the persistence of chronic inflammation further exacerbates cellular senescence [47]. In this context, “nanotanks” not only provide a new strategy for wound healing but also provide the possibility of inhibiting cellular senescence and breaking the cycle of inflammation and aging.

### MMC promote in vivo healing of MRSA infections in diabetic wounds

**Fig. 9.**
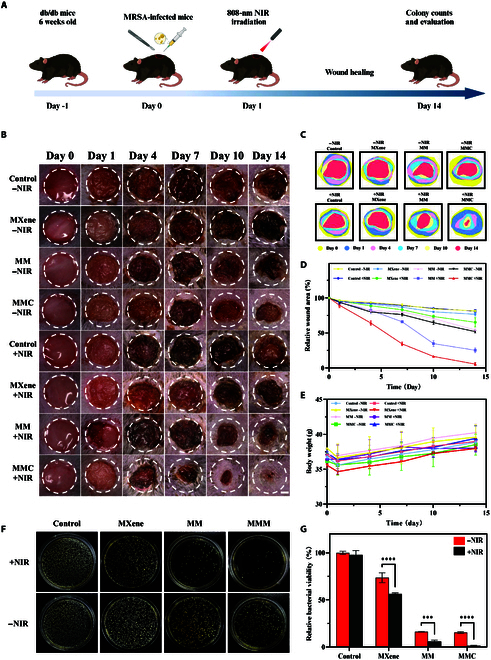
In vivo MRSA-infected wound healing evaluation. (A) Scheme to illustrate the treatment procedure on mouse models with MRSA wound infection. (B) Photographs of MRSA-infected wounds in diabetic mice and (C and D) corresponding statistics of wound closure mice at 0, 1, 4, 7, 10, and 14 d in different treatment groups. (E) Changes in the body weight of mice. (F) Photographs of agar plate bacterial colonies separated from infected wounds of mice in the different treatment groups, together with corresponding statistical analysis of relative bacterial viability (mean ± SEM, *n* = 6, **P < 0.01, ***P < 0.001). (G) Survival rate of bacteria in the wound tissue on the 14th day after treatment.

Based on the outstanding antimicrobial effects of MMC, its positive regulatory effects on inflammation and angiogenesis, and its ability to promote cell migration, we have established a MRSA-infected diabetic full-thickness skin defect model to evaluate its efficacy in the healing of diabetic wounds in vivo. To access the wound healing effects of MMC nanosheets in vivo, we established a wound infection model using MRSA-infected db/db diabetic mice. The MRSA bacterial suspension was inoculated into 8-mm circular full-thickness wounds, and then the mice were randomly divided into 8 groups of 6 mice each: saline, saline +NIR, MXene, MXene +NIR, MM, MM +NIR, MMC, and MMC +NIR. The wound healing process was documented over 14 d across different treatment groups, with representative images shown in Fig. 9B. Over time, the saline control group exhibited minimal wound healing, with subcutaneous pus accumulation, as confirmed by colony plate counts after wound tissue excision (Fig. 9F and G). In contrast, the MXene, MM, and MMC groups displayed improved wound healing when compared to their non-NIR counterparts. Notably, the MMC +NIR group demonstrated the most rapid wound healing, achieving almost complete healing by day 14, suggesting that the synergistic treatment involving CO release from MMC nanosheets notably accelerated wound healing (Fig. 9D).

Throughout the treatment process, there was no statistically significant change in body weight across all groups, indicating minimal impact of the material on body weight (Fig. 9E). Under the irradiation of NIR light, we investigated the temperature changes of skin wounds in mice from the control group and the MMC group (Fig. S4). On day 14, bacteria from skin tissue homogenates were extracted and cultured on agar to evaluate the antimicrobial effect in vivo by counting colony-forming units. The MM +NIR group-treated wounds had a relative colony-forming unit percentage of approximately 5.8%, significantly lower than the control group. The MMC +NIR group had the lowest bacterial count at 1.4%, indicating that wounds treated with MMC +NIR nanosheets were most resistant to infection. This result is consistent with the findings from in vitro antimicrobial experiments.

**Fig. 10.**
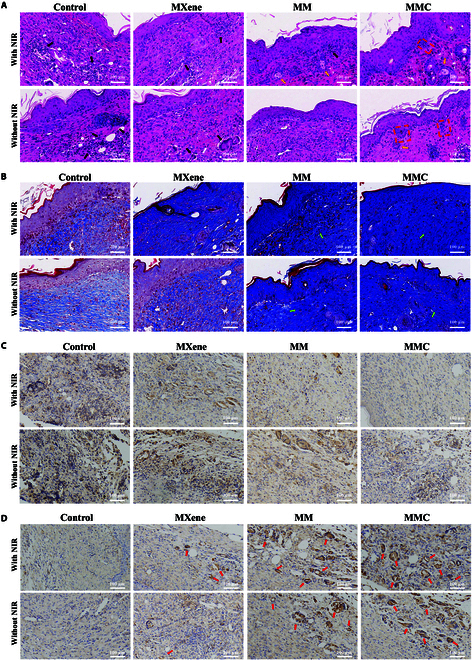
Histological analysis of wound healing. (A) H&E staining images of skin tissues. The black arrows indicate inflammatory cells; the yellow arrows indicate new skin glands; the red rectangular box indicates new epithelial tissue. (B) Masson staining images of skin tissues. The green arrows indicate the generation state of collagen fibers. (C) Immunohistochemical examination for IL-6 in the wound. (D) Immunohistochemical examination for VEGF in the wound. Scale bar: 100 μm.

In diabetic wounds, following bacterial invasion, the toxins released by the infection can lead to the overexpression of inflammatory cytokines. The inflammatory response is not entirely negative; it is an inevitable stage in the tissue repair process [48]. However, an excess of inflammatory cells inhibits wound healing. We performed histologic analysis using H&E staining (Fig. 10A) and Masson’s staining (Fig. 10B) to further evaluate the recovery of infected skin tissue. The histological staining results showed a large inflammatory cell infiltration in the control and MXene groups (indicated by black arrows). In contrast, the MM +NIR and MMC +NIR groups showed a large reduction in the number of infiltrating inflammatory cells due to the combination treatment, along with the appearance of structures resembling normal skin tissue (red boxes), glands (indicated by yellow arrows), and neoplastic hair follicles. Collagen fibers appeared blue after Masson’s staining, and a higher deposition of collagen fibers indicated better wound healing. Similarly, Masson’s staining showed the highest collagen fiber deposition in the MMC +NIR group (indicated by green arrows). Bacteria and their released toxins can cause an overexpression of inflammatory factors, and an excess of these inflammatory factors can also lead to chronic nonhealing wounds. Figure 10C shows the expression of IL-6 in the wound. Quantitative analysis revealed a substantial reduction in IL-6 expression in the MM +NIR and MMC +NIR groups, especially the latter (Fig. S5). Additionally, new blood vessels can provide the nutrients and oxygen required for wound healing, making the number of new blood vessels an important indicator for assessing wound healing. In the immunohistochemical staining for VEGF, the MMC +NIR group showed a substantial increase in angiogenesis (indicated by red arrows) (Fig. 10D). These results collectively highlight the great potential of the treatment strategy based on this “nanotank”, which demonstrates the efficacy of promoting diabetic wound healing through a 3-modality mechanism of PTT, PDT, and CO gas molecule release.

### Biocompatibility evaluation

To more accurately represent clinical conditions, we selected mouse skin fibroblasts (L929), HUVECs, HOKs, and mouse monocyte macrophages (RAW264.7) as in vitro models to assess the potential cytotoxicity of the drug. The CCK-8 assay results indicated the cytotoxicity of MMC nanosheets on L929, HOK, HUVEC, and RAW264.7 at different concentrations (25, 50, 100, 200, and 300 μg/ml). The results showed that at a high concentration of 200 μg/ml, the cell viability of both cell types was still greater than 80%, indicating no obvious cytotoxicity (Fig. 8B and C) (Fig. S6). Additionally, we investigated the cell viability of L929 and HOK cells after treatment with MXene, MM, and MMC groups at a concentration of 100 μg/ml (incubated for 72 h). The results showed that the cell survival rate for both cell types remained above 80% (Fig. 8D and E). In the hemolysis assay, erythrocytes were incubated with different concentrations (25, 50, 100, 200, and 300 μg/ml) of MMC, and PBS and deionized water were used as negative and positive controls, respectively, to ensure the accuracy and reliability of the results. The supernatant of the MMC-treated group was clear, and even at a high concentration (300 μg/ml), the hemolysis rate of MMC was only 0.74%, indicating good blood biocompatibility. This is well below the clinical requirement for the hemolysis rate of biomaterials (<5%) (Fig. 8A).

On the 14th day of treatment, blood was collected from control and MMC-treated mice, and liver and kidney function indices were measured (Fig. S7A to D). We found no significant differences in the levels of urea, uric acid, alanine aminotransferase, aspartate aminotransferase, and albumin, indicating no hepatorenal toxicity.

We collected major organs (heart, liver, spleen, lung, and kidney) from all mice on day 14 after treatment, and H&E staining showed no obvious systemic toxicity of the material to major organs (Fig. S7E). Collectively, these in vivo results demonstrate the high biocompatibility and safety of MMC, confirming its potential for clinical application as an antimicrobial wound healing therapy.

## Conclusion

In this study, a bacterial-targeting “nanotank” was successfully constructed based on MXene, MOF, and CORM-401. The nanotank exhibits a robust antibacterial efficacy through a multifaceted mechanism involving localized photothermal, oxidative damage, and CO release. The 2-dimensional nanomaterial MXene is loaded with ultrasmall particle size MOF, which encapsulates CORM-401. Utilizing the photocatalytic properties of the MOF, it generates ROS under irradiation, and through the ROS cascade, it triggers the rapid release of CO from the encapsulated CORM-401, which is a gas bomb. This innovative approach is based on the synergistic effects of gas therapy, PTT, and PDT. It has been demonstrated to have huge antibacterial effects on drug-resistant bacteria and bacterial biofilms. This 3-modality synergistic treatment strategy markedly accelerates bacterial death and biofilm destruction compared to a single-treatment paradigm. Moreover, the nanoplatform exhibits excellent bacterial targeting capability, which enables precise delivery and control of CO gas release, thus improving the bioavailability and safety of antibacterial drugs. Furthermore, the potent antimicrobial efficacy of this nanoplatform (MMC) was corroborated in a MRSA-infected diabetic mouse wound model, which facilitated the healing of infected wounds without the emergence of drug resistance. This system represents a viable, antibiotic-free therapeutic strategy for the treatment of drug-resistant bacterial infected wounds in future clinical applications. The nanoplatform’s flexible synthesis method, precise targeting, and efficient antimicrobial effect indicate great potential and broad application prospects in the medical field.

## Data Availability

Data will be made available on request.
