# Supplementary material for: Two-Dimensional “Nanotanks” Release “Gas Bombs” through Photodynamic Cascades to Promote Diabetic Wound Healing
Source: Biomater Res. 2024 Oct 29;28:0100. doi: 10.34133/bmr.0100 (PMC11519204; doi:10.34133/bmr.0100)
Supplement: Supplementary 1 — Figs. S1 to S7 [file bmr.0100.f1.zip › Supplemental Material 2.pdf]

Supporting information

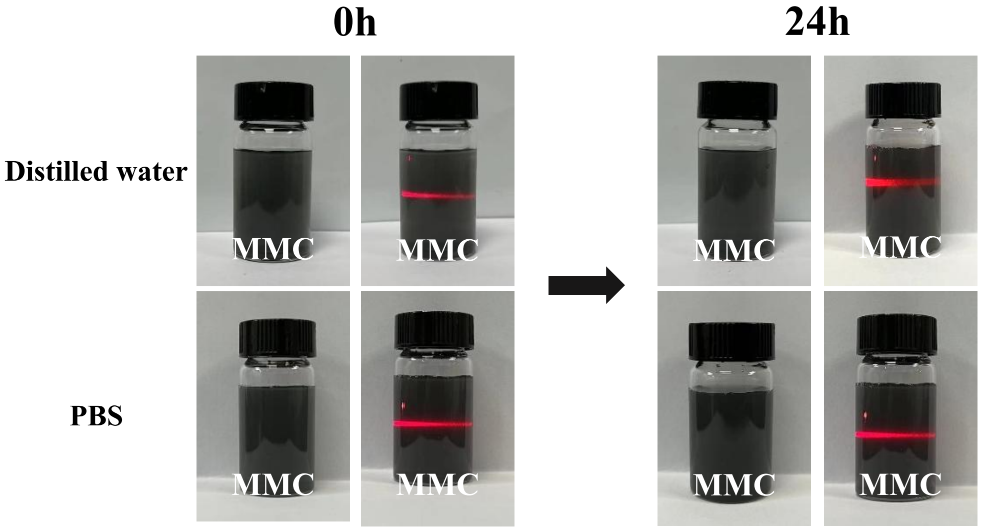

Figure S1. Digital images of MMC colloidal dispersions after standing in distilled water and PBS for 0 and 24 hours.
