# Supplementary material for: Two-Dimensional “Nanotanks” Release “Gas Bombs” through Photodynamic Cascades to Promote Diabetic Wound Healing
Source: Biomater Res. 2024 Oct 29;28:0100. doi: 10.34133/bmr.0100 (PMC11519204; doi:10.34133/bmr.0100)
Supplement: Supplementary 1 — Figs. S1 to S7 [file bmr.0100.f1.zip › Supplemental Material 7.pdf]

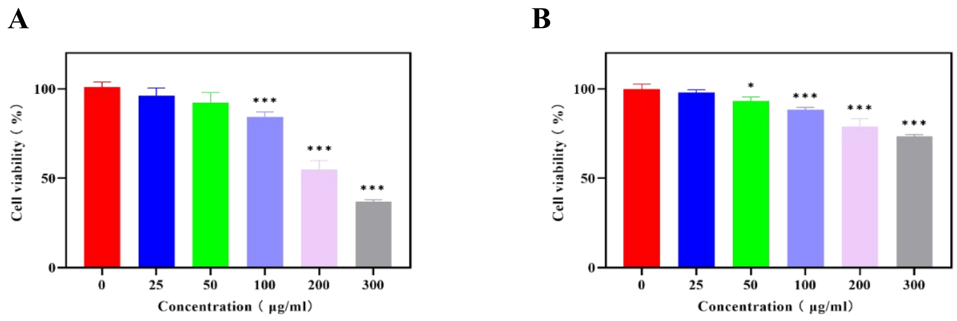

Figure S6. (A, B) Cell viability of HUVEC and RAW264.7 cells after treatment with different concentrations of MMC for 3 days. (mean ± sem, n = 6, **p < 0.01, ***p < 0.001.).
