# Supplementary material for: Two-Dimensional “Nanotanks” Release “Gas Bombs” through Photodynamic Cascades to Promote Diabetic Wound Healing
Source: Biomater Res. 2024 Oct 29;28:0100. doi: 10.34133/bmr.0100 (PMC11519204; doi:10.34133/bmr.0100)
Supplement: Supplementary 1 — Figs. S1 to S7 [file bmr.0100.f1.zip › Supplemental Material 8.pdf]

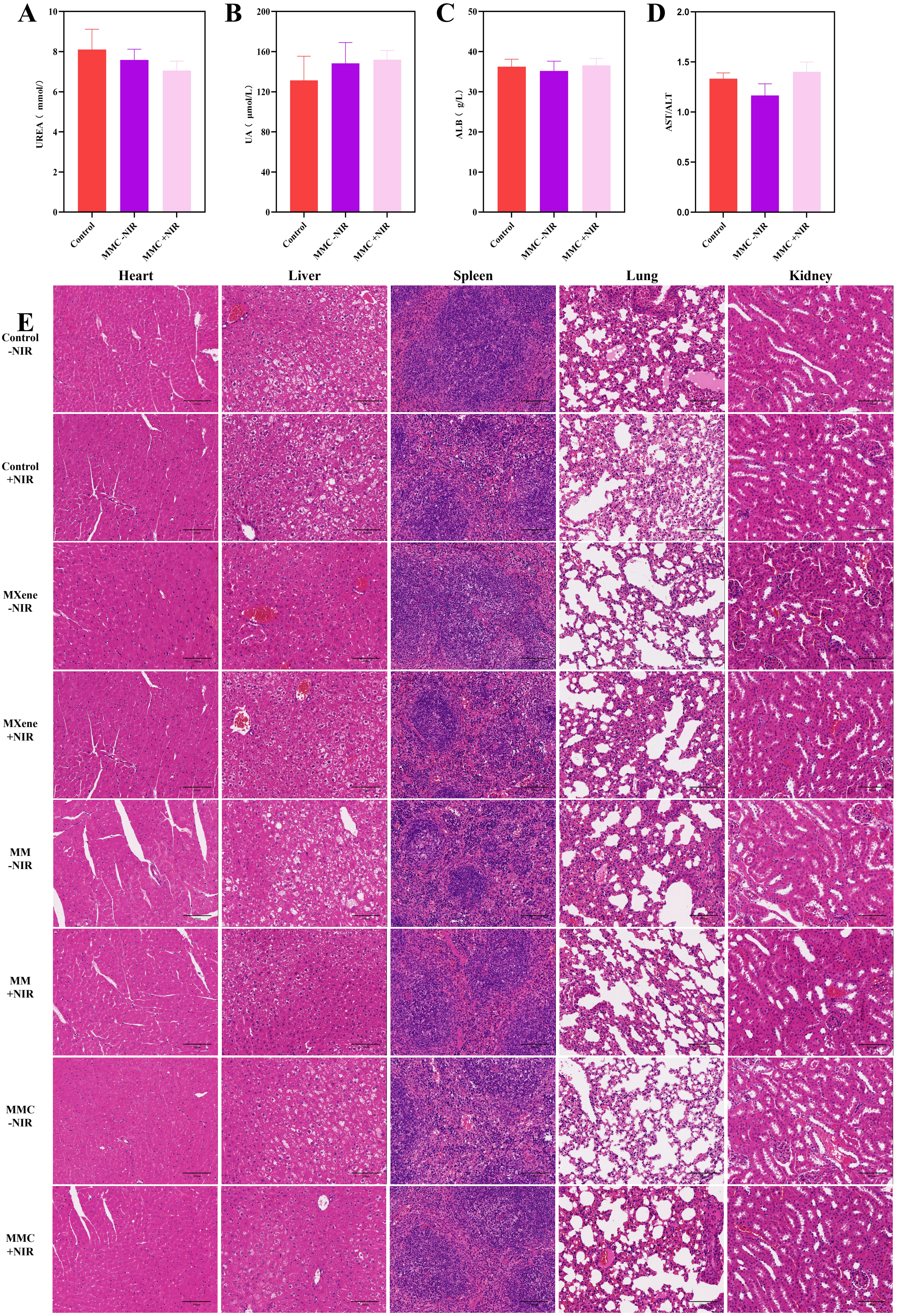

Figure S7. (A-D) Blood serum biochemistry analysis of (A) UREA, (B)UA, (C) ALB and (D) AST/ALT of all mice after 14 days of treatment. (E) H&E staining images of major organs. Scale bars: 100 μm.
