# Supplementary figures and images for: Two-Dimensional “Nanotanks” Release “Gas Bombs” through Photodynamic Cascades to Promote Diabetic Wound Healing
Source: Biomater Res. 2024 Oct 29;28:0100. doi: 10.34133/bmr.0100 (PMC11519204; doi:10.34133/bmr.0100)

**A**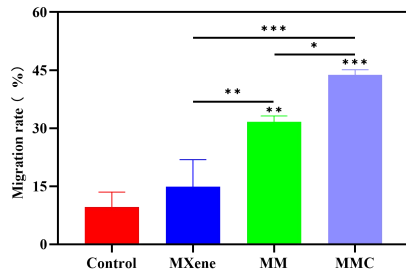**B**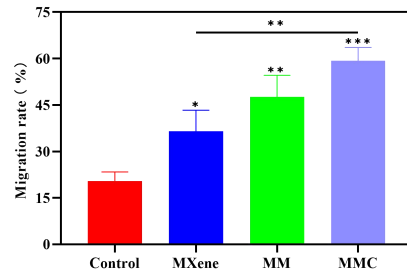

**Figure S3.** (A, B) The migration rate of L929 and HUVEC cells in different treatments (mean  $\pm$  sem, n = 6, \*\*p < 0.01, \*\*\*p < 0.001.).

Supplement: Supplementary 1 — Figs. S1 to S7 [file bmr.0100.f1.zip › Supplemental Material 4.pdf]

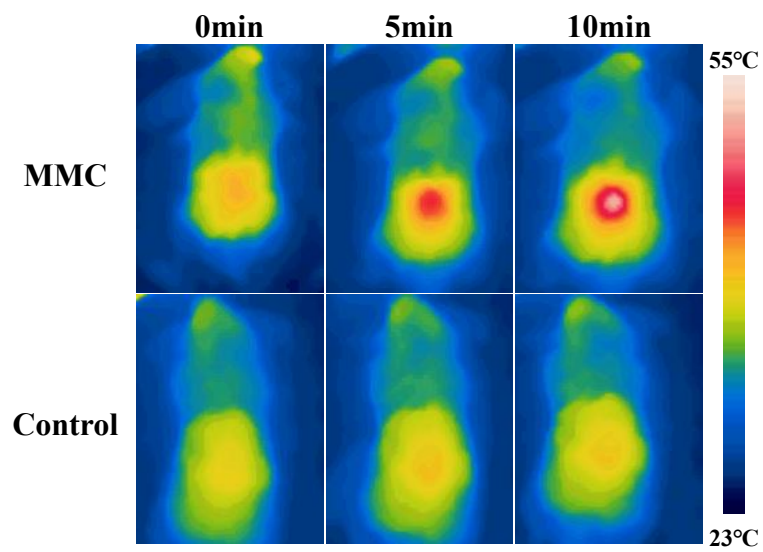

**Figure S4.** The temperature changes of skin wounds in mice from the control group and the MMC group.

Supplement: Supplementary 1 — Figs. S1 to S7 [file bmr.0100.f1.zip › Supplemental Material 5.pdf]
